# HIV Restriction by APOBEC3 in Humanized Mice

**DOI:** 10.1371/journal.ppat.1003242

**Published:** 2013-03-28

**Authors:** John F. Krisko, Francisco Martinez-Torres, John L. Foster, J. Victor Garcia

**Affiliations:** Division of Infectious Diseases, Department of Internal Medicine, Center for AIDS Research, University of North Carolina at Chapel Hill, Chapel Hill, North Carolina, United States of America; Fred Hutchinson Cancer Research Center, United States of America

## Abstract

Innate immune restriction factors represent important specialized barriers to zoonotic transmission of viruses. Significant consideration has been given to their possible use for therapeutic benefit. The apolipoprotein B mRNA editing enzyme catalytic polypeptide 3 (APOBEC3) family of cytidine deaminases are potent immune defense molecules capable of efficiently restricting endogenous retroelements as well as a broad range of viruses including Human Immunodeficiency virus (HIV), Hepatitis B virus (HBV), Human Papilloma virus (HPV), and Human T Cell Leukemia virus (HTLV). The best characterized members of this family are APOBEC3G (A3G) and APOBEC3F (A3F) and their restriction of HIV. HIV has evolved to counteract these powerful restriction factors by encoding an accessory gene designated viral infectivity factor (*vif*). Here we demonstrate that APOBEC3 efficiently restricts CCR5-tropic HIV in the absence of Vif. However, our results also show that CXCR4-tropic HIV can escape from APOBEC3 restriction and replicate *in vivo* independent of Vif. Molecular analysis identified thymocytes as cells with reduced A3G and A3F expression. Direct injection of *vif*-defective HIV into the thymus resulted in viral replication and dissemination detected by plasma viral load analysis; however, *vif*-defective viruses remained sensitive to APOBEC3 restriction as extensive G to A mutation was observed in proviral DNA recovered from other organs. Remarkably, HIV replication persisted despite the inability of HIV to develop resistance to APOBEC3 in the absence of Vif. Our results provide novel insight into a highly specific subset of cells that potentially circumvent the action of APOBEC3; however our results also demonstrate the massive inactivation of CCR5-tropic HIV in the absence of Vif.

## Author Summary

The APOBEC3 family of proteins is a potent cellular defense mechanism capable of restricting a broad range of viruses including HIV. HIV requires a critical accessory protein, Vif, which targets APOBEC3 for degradation thereby shielding its genome from lethal mutagenesis. Previous *in vitro* studies have shown that in the absence of Vif, HIV can be hypermutated by APOBEC3. This potent restrictive function of APOBEC3 has generated strong interest in developing therapeutics based on the APOBEC3/Vif axis. Here we demonstrate *in vivo* that CCR5-tropic HIV can be efficiently restricted by APOBEC3. However, our results also show that CXCR4-tropic HIV can replicate independent of Vif and escape lethal restriction by APOBEC3. Specifically, we show that thymocytes have reduced expression of A3G and A3F and that direct injection of *vif*-defective HIV into the thymus results in viral replication and dissemination. Despite continued Vif-independent HIV replication, the virus remained sensitive to APOBEC3 mutagenesis and was rapidly restricted in tissues with higher A3G and A3F expression. Our results provide novel insight into the restriction of HIV *in vivo* and identify a potentially significant defect in the innate immune defenses that protect the host cell from pathogens.

## Introduction

Innate immune restriction factors embody specialized barriers to zoonotic transmission of viruses. Substantial consideration has been given to their potential use for therapeutic benefit [1], [2]. The apolipoprotein B mRNA editing enzyme catalytic polypeptide 3 (APOBEC3) family of cytidine deaminases are potent innate immune defense factors capable of efficiently restricting endogenous retroelements as well as a diverse range of viruses including Hepatitis B virus, Human Immunodeficiency virus, Human T Cell Leukemia virus, TT virus, and Human Papilloma virus [3]–[8].

The best-characterized APOBEC3 family members are the immune defense molecules APOBEC3G (A3G) and APOBEC3F (A3F) and their lethal restriction of HIV [5], [9]. HIV has evolved to counteract these powerful restriction factors by encoding an accessory gene designated viral infectivity factor (*vif*). *In vitro* studies have elegantly shown that in the absence of Vif, A3G and A3F are encapsidated into nascent virions and deaminate cytosines in the minus strand of HIV DNA during reverse transcription [10]–[12]. APOBEC3 deamination of cytosines in the minus strand of the viral genome occurs at both CC and TC dinucleotide sites, resulting in GG to AG as well as GA to AA mutations in the coding strand of the viral genome [10], [11], [13], [14]. APOBEC3 induced G to A mutations at GG dinucleotide sites are exclusively the result of A3G deamination, while mutations occurring at GA sites can be caused by multiple APOBEC3 proteins including both A3F and A3G [10], [15]. While studies have demonstrated the deleterious effects of G to A hypermutation of the HIV genome [10], [16]–[18], a recent *in vitro* study showed variable levels of A3G induced G to A mutations suggesting that A3G may contribute to viral diversity [19].

In this study, we use humanized mice for the *in vivo* study of HIV in the context of a human immune system. Both NSG-hu and NSG BLT mice are systemically reconstituted with multiple lineages of hematopoietic cells including T cells, B cells, and myeloid cells following transplantation with CD34^+^ hematopoietic stem cells [20], [21]. Additionally, BLT humanized mice are implanted with human liver and thymic tissue under the kidney capsule prior to the transplant of autologous CD34^+^ cells which results in the development of a bona fide human thymus for T cell development [21]. Like any other model for HIV/AIDS research humanized mice have several strengths and limitations that have to be taken into consideration in the development of experimental plan. Two recent review articles cover this area in significant detail [22], [23]. Despite their limitations humanized mouse models have previously been used for the study of HIV transmission, pathogenesis, prevention, therapy and latency/eradication [20], [24]–[28]. Here we first demonstrate the highly effective inactivation of CCR5-tropic HIV-1 by APOBEC3 when unobstructed by a functioning *vif in vivo* after intravenous infection. Secondly, we demonstrate that if injected directly into the thymus, *vif*-defective viruses can replicate escaping absolute APOBEC3 restriction.

## Results

### Mutations in *vif* do not affect virus replication in the absence of APOBEC3

**Figure 1:**
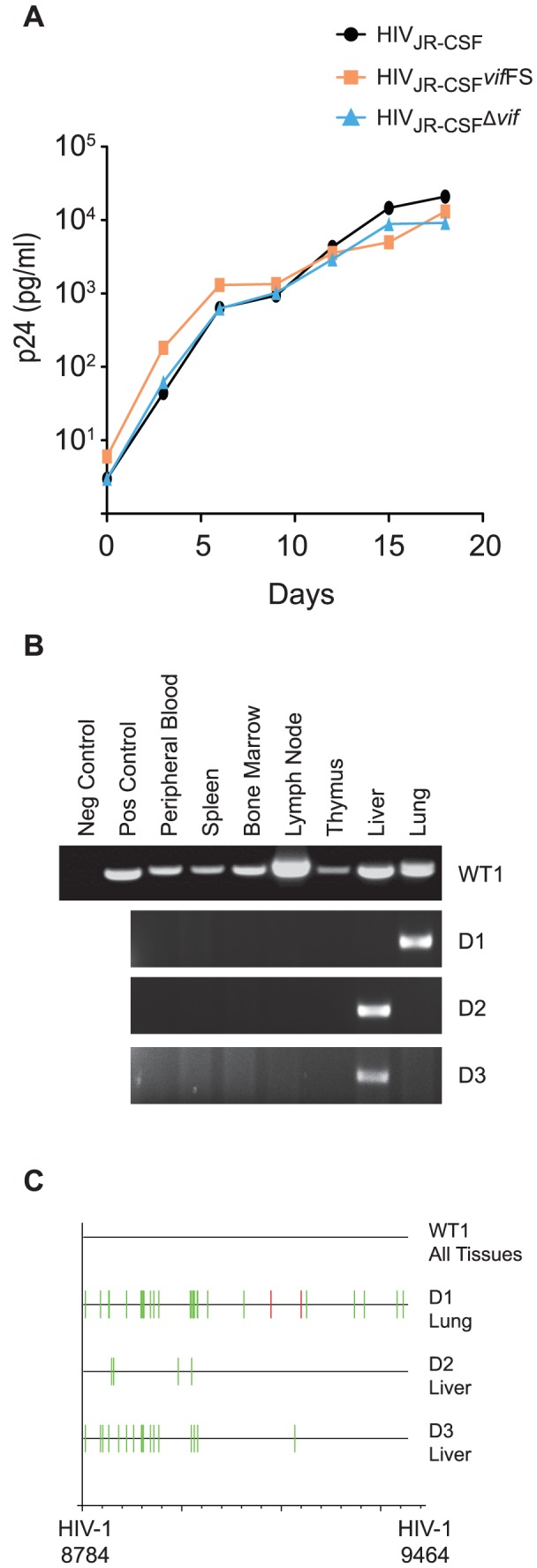
Human APOBEC3 rapidly restricts *vif*-deleted HIV-1_JR-CSF_ *in vivo*. (A) Replication of HIV_JR-CSF_, HIV_JR-CSF_Δ*vif*, and HIV_JR-CSF_ *vif*FS in CEM-SS cells expressing CCR5 (CEM-SS CCR5). Culture supernatant was assayed for p24^Gag^ by ELISA at three day intervals to determine the replication kinetics of the mutant viruses. (B) Nested PCR amplification of viral DNA from the tissues obtained one week post-exposure from a representative NSG-hu mouse infected with 9×10^4^ TCIU of wild-type HIV-1_JR-CSF_ (WT1) or from three mice infected with 3.6×10^5^ TCIU of HIV_JR-CSF_Δ*vif* (indicated as D1–3). (C) Highlighter sequence analysis of 7 wild-type and 3 Δ*vif* HIV DNA sequences. Amplified viral DNA from panel A showed no APOBEC3 induced mutations in HIV_JR-CSF_ (WT1 all sequence from tissues is shown together). In contrast, viral DNA from all positive tissues obtained from HIV_JR-CSF_Δ*vif* infected mice had G to A (green lines) and/or C to T mutations (red lines). HIV-1_JR-CSF_ nucleotide numbers are indicated at the bottom.

To confirm that the mutations disrupting *vif* do not have a detrimental effect on the replicative capacity of HIV-1_JR-CSF_, we generated a CCR5 expressing permissive cell line (CEM-SS CCR5) and infected them with wild-type HIV-1_JR-CSF_ or isogenic viruses containing either an irreparable deletion in *vif* (HIV_JR-CSF_Δ*vif*) or a one base insertion in *vif* (HIV_JR-CSF_ *vif*FS). Replication of both *vif*-defective viruses was equal to that of wild-type in permissive cells, confirming that the disruption of *vif* did not have a deleterious effect on HIV-1 in the absence of APOBEC3 (Figure 1A).

### *vif*-deleted CCR5 tropic HIV-1 administered intravenously is rapidly restricted by APOBEC3 *in vivo*

To assess the *in vivo* effectiveness of APOBEC3 restriction of HIV, we intravenously infected NSG-hu mice with wild-type HIV-1_JR-CSF_ (a T-cell CCR5-tropic primary isolate) or HIV_JR-CSF_Δ*vif*. As early as one week after intravenous infection, widespread replication of wild-type virus was detected as HIV DNA was amplified from every tissue analyzed (Figure 1B). In contrast the *vif*-defective virus did not sustain replication in humanized mice; as viral DNA was sparsely present (Figure 1B). Notably, HIV_JR-CSF_Δ*vif* DNA could only be amplified from one organ from each infected mouse suggesting that an extremely low number of infected cells were present. Analysis of the viral DNA sequence from the animals revealed that HIV DNA from mice infected with wild-type virus had no mutations, whereas viral DNA from the HIV_JR-CSF_Δ*vif* infected mice had numerous G to A mutations consistent with APOBEC3 induced restriction (Figure 1C). The limited number of tissues with cells harboring G to A mutated HIV_JR-CSF_Δ*vif* DNA one week after exposure suggests that APOBEC3 restriction of *vif*-defective HIV occurs rapidly *in vivo*.

### APOBEC3 restriction of CCR5 tropic in the absence of *vif*

**Figure 2:**
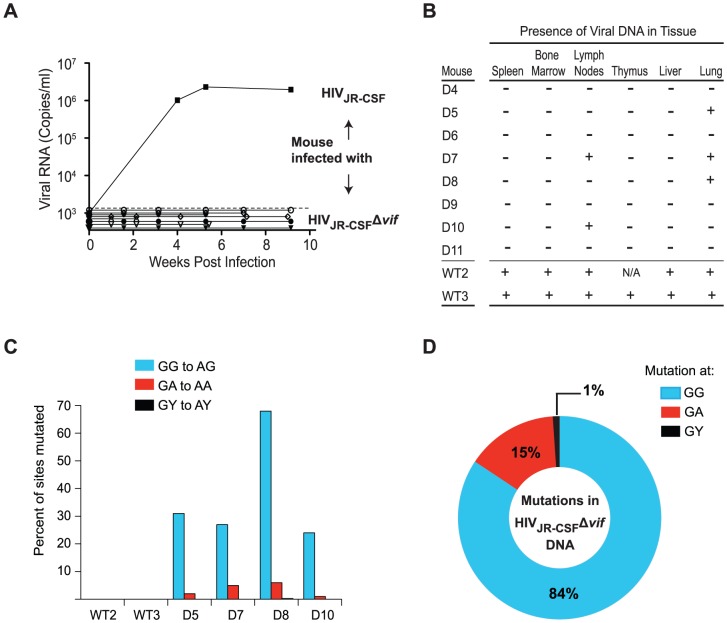
*vif*-deleted HIV-1_JR-CSF_ does not overcome APOBEC3 restriction *in vivo*. (A) Longitudinal analysis of plasma viral load in humanized mice intravenously infected with 9×10^4^ TCIU of wild-type HIV-1_JR-CSF_ or 3.6×10^5^ TCIU of HIV_JR-CSF_Δ*vif*. (B) Detection of HIV DNA (+) by nested PCR from the tissues of humanized mice in panel A. Negative tissues (−) yielded no amplified viral DNA using two independent nested PCR primer sets targeting separate regions of the viral genome. N/A = not analyzed. (C) Percentage of putative APOBEC3 mutation sites (GG, GA, GY) that were mutated in 17 viral DNA sequences amplified from the tissues of infected mice. Viral DNA from mice infected with HIV_JR-CSF_Δ*vif* had 25%–65% of GG sites mutated. (D) G to A mutational profile of all viral DNA from mice infected with HIV_JR-CSF_Δ*vif*. Percentages indicate the proportion of G to A mutations occurring at GG (blue), GA (red), or GY (black) sites. D4-D8, WT2-WT3, NSG-hu mice. D9-D11, NSG-BLT mice.

While evidence of APOBEC3 restriction of *vif*-deficient HIV is observed early after infection, we next determined whether HIV_JR-CSF_Δ*vif* could develop resistance to APOBEC3 and replicate systemically. To address this, we infected humanized mice (n = 8) intravenously with HIV_JR-CSF_Δ*vif* and monitored them for plasma viral load. Longitudinal analysis demonstrated that HIV_JR-CSF_Δ*vif* restriction by APOBEC3 is absolute, as no viral RNA was present in the plasma of the mice at any time point in contrast to infection with wild-type HIV_JR-CSF_ (Figure 2A). No revertants or complementary changes arose that restored the ability of HIV_JR-CSF_Δ*vif* to replicate. In contrast to the widespread presence of viral DNA in all tissues analyzed after wild-type HIV infection, the extremely limited replication of HIV_JR-CSF_Δ*vif* was confirmed by the absence of HIV DNA in tissues obtained from 4/8 infected mice, and the presence of lethally mutated viral DNA in only a few tissues of the other four animals (Figure 2B). To determine the extent of APOBEC3 hypermutation in the HIV_JR-CSF_Δ*vif* provirus, we analyzed the sequences for G to A mutations at GG, GA and GY dinucleotide sites which are the targets of the APOBEC3 proteins. We found that 25–65% of the GG sites had been mutated with additional (albeit fewer) mutations present at GA sites, demonstrating extensive APOBEC3 hypermutation to lethally restrict HIV_JR-CSF_Δ*vif* (Figure 2C). Analysis of the mutational profile in the HIV_JR-CSF_Δ*vif* DNA from the mice showed that 84% of all the G to A mutations occurred at GG dinucleotide sites whereas only 15% of mutations were present at GA sites and only 1% occurred at GY sites (Figure 2D). Taken together, these results demonstrate that HIV_JR-CSF_Δ*vif* is unable to overcome the loss of Vif and is lethally restricted by APOBEC3 *in vivo*.

### Human APOBEC3 exerts a strong selective pressure on HIV-1 *in vivo*

**Figure 3:**
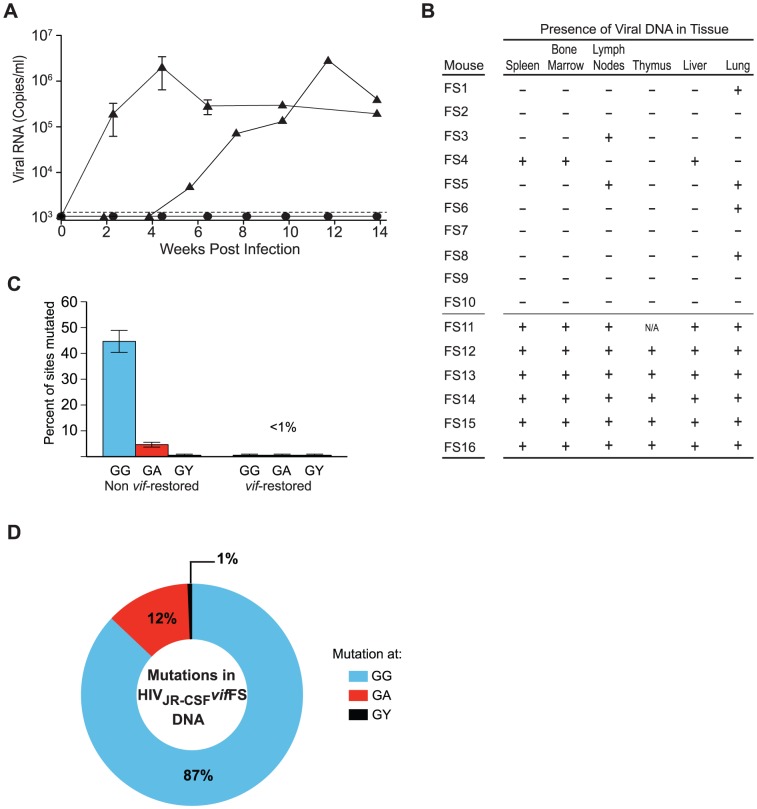
Human APOBEC3 exerts a strong selective pressure on HIV-1_JR-CSF_ containing a frameshift in *vif*. (A) Plasma viral load analysis in humanized mice intravenously infected with 9×10^4^ or 3.6×10^5^ TCIU of HIV_JR-CSF_ *vif*FS. Viral RNA was not detected in the plasma (circles, n = 10) unless the *vif* ORF is restored (triangles, n = 6). The appearance of plasma viremia was delayed by 4 weeks in one of these mice. (B) Detection of HIV DNA (+) by nested PCR from the tissues of humanized mice intravenously infected with HIV_JR-CSF_ *vif*FS. Negative tissues (−) yielded no amplified viral DNA using two independent nested PCR primer sets targeting separate regions of the viral genome. Viral DNA is sparsely present in tissues from mice where *vif* was not restored (indicated as FS1–10). In contrast, all tissues analyzed from the six mice where *vif* was restored had viral DNA present (FS11–16). N/A = not analyzed. (C) Percentage of putative APOBEC3 mutation sites (GG, GA, GY) that were mutated in 76 viral DNA sequences amplified from the tissues of HIV_JR-CSF_ *vif*FS infected mice where *vif* was not restored (40% of all GG sites mutated) or mice where *vif* was restored (no hypermutation). Data represent mean +/− SEM. (D) G to A mutational profile of all viral DNA from mice infected with HIV_JR-CSF_Δ*vif*. Percentages indicate the proportion of G to A mutations occurring at GG (blue), GA (red), or GY (black) sites. FS1–FS9, FS11–FS14, NSG-hu mice. FS10, FS15–FS16 NSG-BLT mice.

To evaluate the selective pressure exerted by APOBEC3 on HIV *in vivo*, we used a mutant isogenic virus containing a one base insertion in *vif* (HIV_JR-CSF_ *vif*FS) to intravenously infect 16 humanized mice representing 7 different human donors. Consistent with our previous results, in 10/16 mice intravenously infected with HIV_JR-CSF_ *vif*FS there was no evidence of virus replication as determined by the absence of viral RNA in the plasma (Figure 3A). However, in the remaining six mice the virus was able to replicate to levels similar to those observed with the wild-type virus (Figure 3A). One salient aspect noted was the almost complete absence of APOBEC3 mutations in viral RNA samples obtained from the plasma from these six mice. Molecular analysis of viral sequences from the peripheral blood from these mice demonstrated that in all six cases a one-nucleotide deletion had occurred that fully restored the *vif* open reading frame (ORF) highlighting the extreme selective pressure APOBEC3 exerts on HIV *in vivo* to restore Vif activity or be lethally mutated. Two important issues that should be noted are 1) the virus used for these experiments was generated via transient transfection of 293T cells creating a uniform inoculum and 2) that this repair mutation occurring *in vivo* is not at a putative APOBEC3 site and therefore it is most likely is a result of a mutation occurring during reverse transcription.

We then analyzed viral DNA from the tissues of all 16 mice exposed to HIV_JR-CSF_ *vif*FS. In multiple tissue samples from four of the aviremic mice we found no evidence of HIV DNA. In similar samples from six other aviremic mice only low levels of heavily mutated HIV DNA was present in a few tissues (30–60% of the GG dinucleotide sites mutated) (Figure 3B and 3C). The mutational profile of the viral DNA from these animals again showed a preference for GG sites accounting for 87% of all mutations (Figure 3D). In sharp contrast, in mice where the *vif* ORF was restored virtually intact viral DNA was present in every tissue analyzed, highlighting the strong selective pressure exerted by APOBEC3 on HIV (Figure 3B and 3C). The lack of G to A mutations in the *vif*-restored viral genome suggested that the virus had evaded APOBEC3 restriction until restoring *vif*.

### HIV-1_JR-CSF_ *vif*FS restores *vif* following direct injection into the thymus

**Figure 4:**
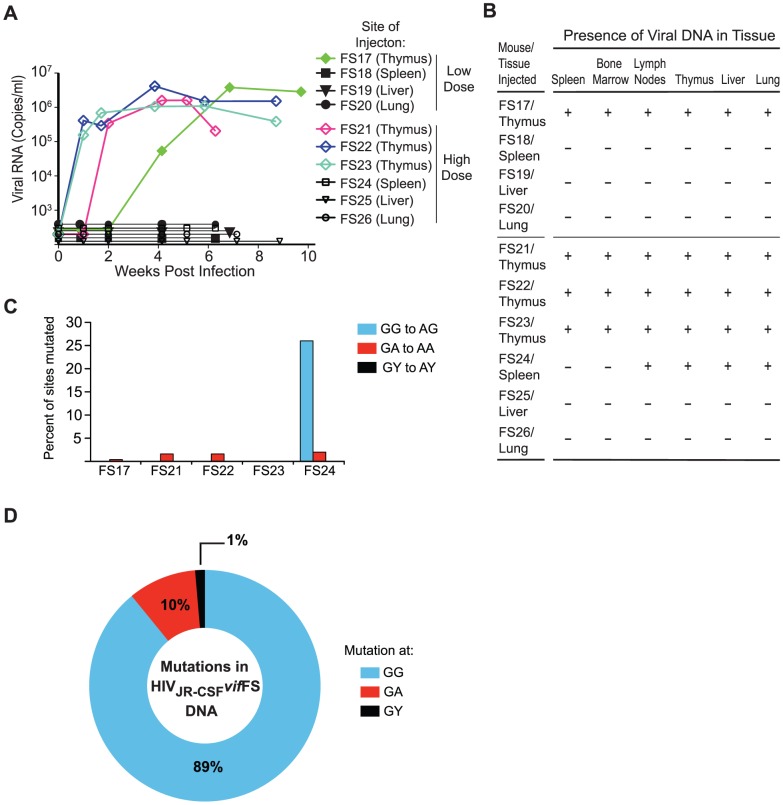
Restoration of *vif* occurs only following direct virus injection into the thymus. (A) Longitudinal analysis of plasma viral load in NSG-BLT humanized mice infected with HIV_JR-CSF_ *vif*FS directly into the human thymic implant, spleen, liver, or lung. Solid symbols represent mice infected with a low dose of virus (9×10^4^ TCIU), open symbols represent the high dose infection (3.6×10^5^ TCIU). (B) Detection of HIV DNA (+) by nested PCR from the tissues of humanized mice in panel A. Negative tissues (−) yielded no amplified viral DNA using two independent nested PCR primer sets targeting separate regions of the viral genome. (C) Percentage of putative APOBEC3 mutation sites (GG, GA, GY) that were mutated in 44 viral DNA sequences amplified from the tissues of mice injected with HIV_JR-CSF_ *vif*FS. Mice injected into the thymic implant had no GG to AG mutations in any tissue whereas HIV DNA from four tissues of mouse FS24 injected into the spleen is hypermutated. (D) G to A mutational profile of all viral DNA from mouse FS24 which failed to restore the *vif* ORF. Percentages indicate the proportion of G to A mutations occurring at GG (blue), GA (red), or GY (black) sites.

The stochastic nature of *vif* ORF restoration may reflect its occurrence in a specific anatomical location(s). Therefore we directly injected 9×10^4^ TCIU of HIV_JR-CSF_ *vif*FS into the spleen, liver, lung or human thymic implant of separate humanized mice. Evidence of viral replication in peripheral blood was exclusively found when HIV_JR-CSF_ *vif*FS was injected directly into the thymus (Figure 4A). In this case, sequence analysis of *vif* showed a one base deletion restoring Vif expression. Injection of the virus into the spleen, liver, or lung resulted in absolute restriction with no viremia and no residual viral DNA present in any tissue (Figure 4A and 4B)

To determine if restoration of the *vif* ORF following thymic injection was non-random, we increased the virus inoculum four-fold and repeated the infection, using 3.6×10^5^ TCIU of HIV_JR-CSF_ *vif*FS injected into the same set of tissues. Again evidence of HIV replication was only observed after intrathymic exposure with 3/3 mice that received the virus directly into the thymus becoming viremic (Figure 4A). Strikingly, despite the high virus inoculum injected directly into the spleen, liver, or lung no evidence of virus replication was observed (Figure 4A). When tissues from these animals were analyzed, we found that only one mouse, FS24, had viral DNA present (Figure 4B) and that it had all been lethally mutated by APOBEC3 (Figure 4C and 4D). These results demonstrate that transient HIV_JR-CSF_ *vif*FS replication and subsequent *vif* restoration specifically occurs following a direct thymic exposure. Furthermore, the potent antiretroviral activity of APOBEC3 is highlighted by the absolute restriction of HIV_JR-CSF_ *vif*FS when the virus is injected into other tissues.

### APOBEC3G and APOBEC3F expression is reduced in the thymus

**Figure 5:**
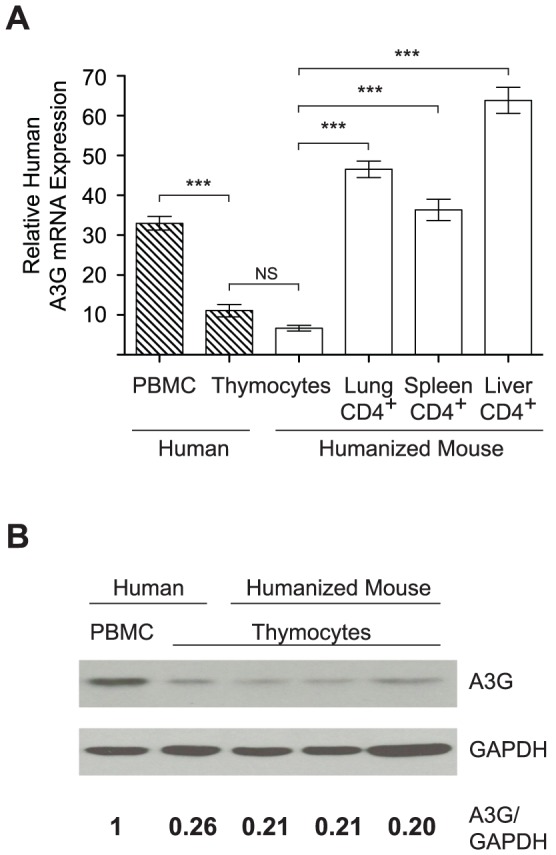
APOBEC3G expression is reduced in the thymus. (A) Human A3G mRNA levels from human PBMC (n = 7), human thymus (n = 6), NSG-BLT humanized mouse thymus (n = 18), and CD4^+^ cells from NSG-BLT humanized mouse lung (n = 6), spleen (n = 7), liver (n = 7) were determined using qRT-PCR and normalized to human TATA Box binding protein. Hatched bars represent human cells; open bars represent humanized mouse cells. NS =  not significant, *** p<0.01 by one way ANOVA with Bonferroni's posttest. Data represent mean +/− SEM. (B) Immunoblot for human A3G and GAPDH from human PBMC and cells from human and humanized mouse thymus. Numbers represent A3G normalized to GAPDH compared to human PBMC which is set to 1.

Since the reversion of the *vif* ORF specifically occurred following injection of the virus into the thymus, we next determined whether A3G and A3F expression was lower in the thymus compared to other tissues. We tested this by comparing A3G and A3F mRNA levels in purified thymocytes (of which >90% are CD4^+^) with those in CD4^+^ cells isolated from other tissues in humanized mice. Our results show that thymocytes express 4–8 fold less A3G mRNA and 2.5–3.5 fold less A3F mRNA than human CD4^+^ cells isolated from the spleen, liver or lung (Figure 5A and Figure S1). Furthermore, no difference in A3G or A3F mRNA expression was found in thymocytes from humanized mice or human thymus. Additionally, A3G in thymocytes was found to be 3–4 fold lower compared to human peripheral blood mononuclear cells (PBMC) by both mRNA and protein expression (Figure 5A and 5B). These results are consistent with the observation that *vif* reversion specifically occurs following thymic injection of HIV_JR-CSF_ *vif*FS and suggests that the thymus may support Vif-independent HIV replication.

Based on our observations of reduced A3G and A3F expression in thymocytes and reversion of the *vif* ORF exclusively occurring with thymic exposure, we considered the possibility that a direct injection of HIV_JR-CSF_Δ*vif* (the virus containing a non-revertible deletion in *vif*) into the thymus would result in Vif-independent replication. HIV RNA was transiently observed in the plasma of 2/6 mice following thymic infection with this virus (Figure S2). This low level of replication in some mice is consistent with the results presented above with frame shift containing HIV_JR-CSF_ *vif*FS (Figure 4A), in which the virus had restored the *vif* ORF and was able to replicate unimpeded by APOBEC3 after reversion. These results show that recovery of Vif activity is necessary for ongoing replication and viral dissemination by *vif*-defective HIV_JR-CSF_.

### Vif-independent CXCR4-tropic HIV-1 replication is sustained *in vivo*

**Figure 6:**
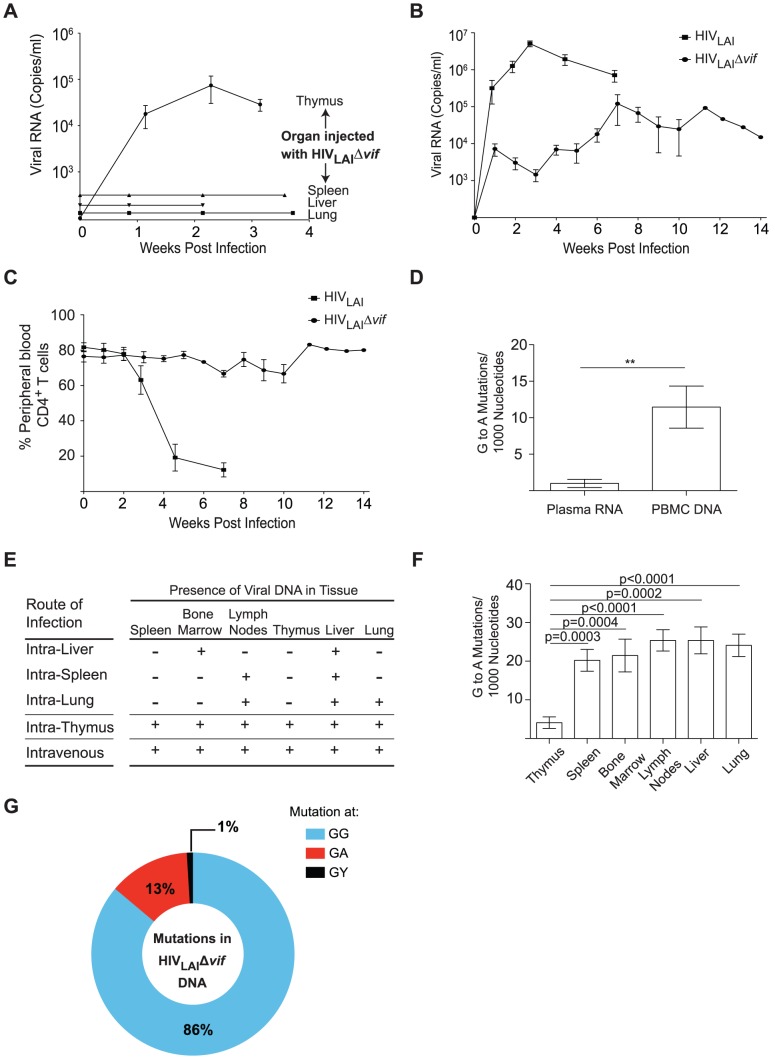
Sustained Vif-independent replication of CXCR4 tropic HIV-1. (A) Plasma viral load was monitored in NSG-BLT humanized mice infected with 3.6×10^5^ TCIU HIV_LAI_Δ*vif* directly into the human thymic implant, spleen, liver, or lung. Direct injection of HIV_LAI_Δ*vif* into the thymus resulted in plasma viremia in 4/4 infections. (B) Longitudinal analysis of plasma viral load in humanized mice infected intravenously with 3.6×10^5^ TCIU of HIV_LAI_Δ*vif* (n = 7) or 3–9×10^4^ TCIU of wild-type HIV_LAI_ (n = 6). Data represent mean +/− SEM. (C) Longitudinal analysis of the percentage of CD4^+^ T cells in the peripheral blood of humanized mice infected in panel B. (D) Comparison of the G to A mutation frequency in the viral RNA from the plasma and the viral DNA from peripheral blood cells from mice intravenously infected with HIV_LAI_Δ*vif*. Data represent mean +/− SEM from 18 sequences, ** p = 0.0066. (E) Detection of HIV DNA (+) by nested PCR from the tissues of BLT humanized mice in panels A and B. Negative tissues (−) yielded no amplified viral DNA using two independent nested PCR primer sets targeting separate regions of the viral genome. Direct injection of HIV_LAI_Δ*vif* into the liver, lung or spleen resulted in limited tissue distribution of viral DNA. (F) Comparison of the G to A mutation frequency in viral DNA from the thymus compared to viral DNA from other tissues of mice infected intrathymically (n = 4) and intravenously (n = 7) with HIV_LAI_Δ*vif*. Data represent mean +/− SEM from 83 sequences. (G) G to A mutational profile of all viral DNA from mice infected with HIV_LAI_Δ*vif*. Percentages indicate the proportion of G to A mutations occurring at GG (blue), GA (red), or GY (black) sites.

We hypothesized that the lack of robust and sustained replication of HIV_JR-CSF_Δ*vif* following direct thymic infection could be due to limited CCR5 expression in the thymus, as <5% of thymocytes express CCR5 whereas 30–40% of thymocytes express CXCR4 [29]–[31]. We therefore introduced the deletion described above into the *vif* ORF of HIV-1_LAI_, a CXCR4-tropic virus (HIV_LAI_Δ*vif*) and confirmed that the disruption of *vif* did not affect the ability of the virus to replicate in the absence of APOBEC3 (Figure S3). We tested our hypothesis by directly injecting HIV_LAI_Δ*vif* into the thymus of four humanized mice. Viremia was present in 4/4 animals inoculated in this manner (Figure 6A). In contrast, when HIV_LAI_Δ*vif* was directly injected into the spleen, liver, or lung of an additional 3 animals viral replication was absolutely restricted (Figure 6A). These results further demonstrate that Vif-independent HIV replication can be sustained following exposure into the thymus but is vigorously restricted in other tissues. Additionally, when HIV_LAI_Δ*vif* was injected intravenously, sustained levels of viral replication were observed in the plasma of humanized mice; however this replication was lower relative to the parental virus (Figure 6B). Remarkably, unlike wild-type HIV_LAI_ which rapidly depletes peripheral blood CD4^+^ T cells, infection with HIV_LAI_Δ*vif* did not deplete CD4^+^ T cells in the periphery despite sustained viral replication (Figure 6C). Sequencing of viral RNA obtained from the plasma of HIV_LAI_Δ*vif* infected mice showed significantly fewer G to A mutations compared to the same region of viral DNA isolated from PBMC, suggesting that the infection was likely being sustained in cells with lower APOBEC3 expression (Figure 6D).

**Figure 7:**
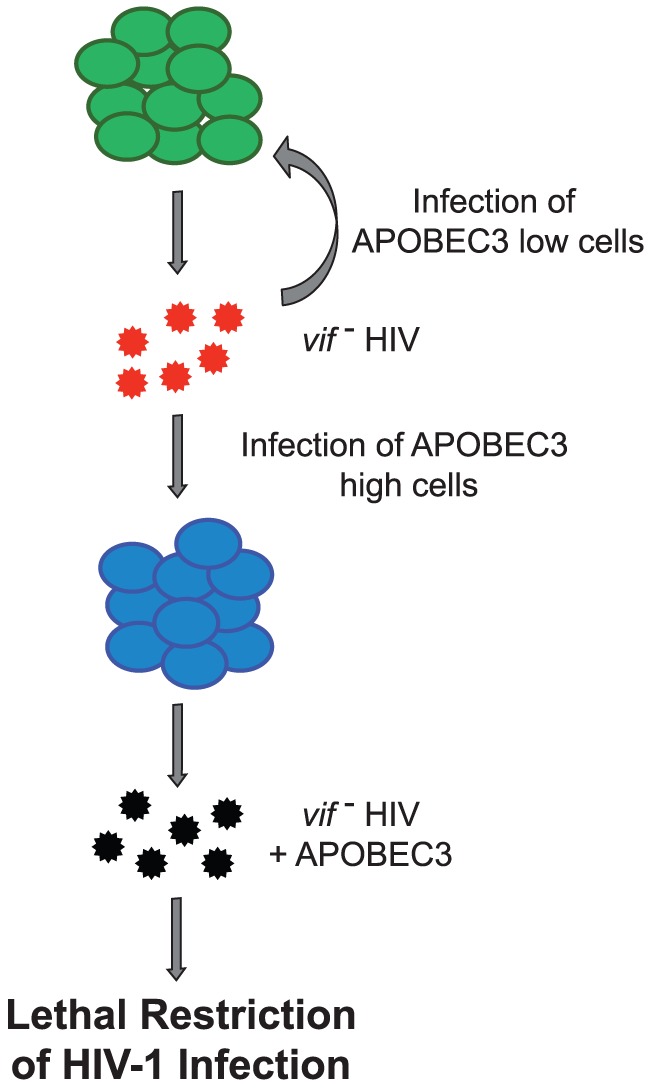
Model depicting sustained *in vivo* replication of HIV_LAI_Δ*vif*. Ongoing viral replication persists in cells with low APOBEC3 (shown in green) with little to no APOBEC3 mutation but infection of cells expressing high APOBEC3 (shown in blue) leads to hypermutation of the viral genome, resulting in lethal restriction of HIV. Replication competent viruses are shown in red, defective virions are shown in black.

Consistent with these results, HIV_LAI_Δ*vif* DNA was abundant in the tissues of intrathymically or intravenously exposed mice while direct exposure into the spleen, liver, or lung resulted in viral DNA sparsely present in the organs (Figure 6E). Since this virus could not restore Vif expression, its viral DNA had G to A mutations; however, consistent with the low expression of A3G and A3F in the thymus (Figure 5A, 5B and S1), significantly fewer G to A mutations were present in viral DNA amplified from the thymus when compared to the same region of the viral DNA amplified from other organs (Figure 6F). Analysis of the mutational profile in the HIV_LAI_Δ*vif* DNA from the mice showed that 86% of all the G to A mutations occurred at GG dinucleotide sites (Figure 6G). The presence of hypermutated provirus in several tissues suggests that the HIV was not able to develop resistance to APOBEC3 by second site mutations in the absence of *vif*, but was instead persisting in a pool of cells that permitted replication (Figure 7). Taken together, these results demonstrate that HIV can sustain replication independent of *vif* escaping APOBEC3 restriction *in vivo*.

## Discussion

Our results demonstrate evidence of *vif*-independent replication of HIV in an *in vivo* setting. The observation that infection with CCR5 tropic HIV is rapidly extinguished in the absence of *vif* while CXCR4 tropic HIV lacking *vif* can sustain replication suggests that *vif*-independent HIV replication is occurring in a location with a paucity of cells expressing CCR5, such as the thymus where <5% of cells express CCR5 while a far greater number of cells (30–40%) express CXCR4 [29]–[31]. Consistent with this observation, direct injection of *vif*-deficient HIV into organs resulted in detectable viral replication only following thymic infection. Remarkably, reversion of the *vif* ORF with HIV_JR-CSF_ *vif*FS occurred in 100% of thymic exposures while being absolutely restricted when injected into all other organs, highlighting the potent antiretroviral activity of APOBEC3. Interestingly, despite the strong selective pressure applied by APOBEC3, we did not observe any evidence of *vif*-defective HIV-1_JR-CSF_ altering its coreceptor usage to take advantage of the lower A3G and A3F expression in the thymus. Coreceptor switching is a complicated process involving multiple mutations in envelope that occurs over a period of years in patients [32]. During their short lifespan, coreceptor switching is not common in humanized mice and has only been reported in a single mouse [33].

Our results demonstrate massive inactivation of CCR5-tropic HIV-1 when the protective effects of Vif are absent. These results are consistent with previously published work by Sato et. al. [34]. Under their experimental conditions, these investigators found that *vif*-defective CCR5-tropic HIV did not replicate at all in humanized mice. Replication of HIV-1 with a functional *vif* gave a different result. In this case, there was a low level of G to A mutation in both A3G and A3F contexts in viral DNA sequences [34]. Thus, these authors confirmed in the humanized mouse model the early observations of the occasional occurrence of hypermutation of HIV-1 isolated from patients [35]–[38].

Analysis of HIV DNA in aborted infections for G to A hypermutation, the hallmark of APOBEC3 restriction, demonstrated that when HIV DNA was present, there was an overwhelming prevalence of mutations at GG dinucleotide sites indicating that in the absence of *vif* A3G is the dominant HIV restricting factor *in vivo* [10]–[12]. This conclusion is further supported by two recent papers using stably expressed A3F or gene targeting to create null mutants to systematically disrupt the individual APOBEC3 proteins that have elegantly demonstrated that A3G is the APOBEC3 family member that induces the preponderance of GG to AG mutations in *vif*-deficient HIV DNA [15], [39]. One substantial benefit of A3G restriction is that the mutation of GG to AG can be highly effective in inactivating viral genes because of the conversion of tryptophan codons (TGG) to stop codons (TAG, TGA, or TAA).

The lower level of GA to AA mutations that we observed suggests a contributory role for A3F in the overall level of G to A mutations we observed. The impact of A3F remains unclear however since the specificity of A3G for the GG context is not absolute and some of the GA to AA mutations we observed may have been created by A3G. A role for A3F in HIV restriction has been questioned recently but this issue remains unresolved *in vivo* [39], [40]. Future experiments with humanized mice will address this question.

The results presented here demonstrate that *in vivo* HIV fails to develop second-site mutations to compensate for the absolute loss of *vif* to overcome A3G induced mutation, which is in contrast to observations made with *in vitro* systems with ectopically expressed A3G [41], [42]. This potent restriction of HIV *in vivo* is not observed by inactivation of other HIV-1 accessory genes [43], [44]. To survive *in vivo* in the absence of *vif*, HIV relies on target cells with reduced A3G expression in which it can replicate as shown by the lack of G to A hypermutation in the cell free virus despite the abundance of G to A mutations present in viral DNA in several tissues with high levels of A3G expression. Our analysis CD4^+^ cells identified thymocytes as a cell population that has reduced A3G expression. Previous analysis of A3G expression from whole tissues did not identify thymocytes as having reduced A3G; however these results are difficult to interpret because of the lower percentage of CD4^+^ cells in organs other than the thymus [45], [46]. Furthermore, the significance of A3G expression levels in the modulation of both wild-type and *vif*-deficient HIV replication has been previously demonstrated in Th1 and Th2 cells [47].

The implications of our findings might not be limited to HIV. Rather they might also extend to other viruses and retroelements that are restricted by APOBEC3 proteins [3], [4], [6]–[8], [48], as they may also persist as a result of reduced APOBEC3 expression that affords them the opportunity to replicate. The expansive restricting activity of the APOBEC3 family on endogenous and exogenous retroviruses serves to illustrate the broad therapeutic implications of our observations. This study also raises an important issue that must be addressed if the Vif-APOBEC3 axis is to be used to develop small molecular inhibitors of HIV replication: the well-documented ability of HIV to develop resistance to all current antiretroviral drugs. By incorporating point mutations in the relevant viral genes HIV can develop drug resistance [49]. Our observation of Vif-independent replication after direct injection into the thymus are consistent with previous work in humanized mice [50] and highlight the potential for HIV to escape the effect of a therapeutic Vif inhibitor [51]–[53]. The drug resistant virus would then be capable of systemic dissemination. However, as with other antiretrovirals, the use of combination therapy may prevent the emergence of such resistance. The thymus plays a critical role in HIV infection as it is actively involved in immune reconstitution following suppression of viremia with antiretroviral therapy. While this immune reconstitution occurs better in children than in adults, extensive thymic damage and incomplete virus suppression hinder this process [54]–[56].

Finally, it remains to be established if sublethal restriction by other innate immune defense proteins such as Tetherin, Trim-5-alpha, SamHD1, etc. could allow the replication of other pathogenic viruses [1]. Therefore, our discovery has long lasting implications that provide an alternative view of the dynamic interplay between endogenous immune restriction factors and the broad spectrum of pathogens they control.

## Materials and Methods

### Ethics statement

All animal experiments were conducted following NIH guidelines for housing and care of laboratory animals and in accordance with The University of North Carolina at Chapel Hill (UNC-Chapel Hill) in accordance with protocols approved by the institution's Institutional Animal Care and Use Committee. UNC-Chapel Hill protocol number 12-170.

### Proviral constructs, virus stocks and cell lines

Experiments were performed using the CCR5-tropic primary isolate HIV-1_JR-CSF_ (accession # M38429) or the CXCR4-tropic molecular clone HIV-1_LAI_ (accession # K02013) [57], [58]. Mutations disrupting *vif* were made in regions that did not affect the overlapping 3′ terminus of *pol* or the splice acceptor site of *vpr*. A non-revertible 172 nucleotide deletion in the 5′ half of HIV-1_JR-CSF_ *vif* (HIV_JR-CSF_Δ*vif*) was constructed by deleting nucleotides 5138 to 5309 between the NdeI and NcoI sites. A second HIV-1_JR-CSF_ with a potentially revertible *vif* (HIV_JR-CSF_ *vif*FS) was constructed by inserting a single adenosine after nucleotide 86 in *vif* by site directed mutagenesis. A non-revertible 178 nucleotide deletion in the 5′ half of HIV-1_LAI_ *vif* (HIV_LAI_Δ*vif*) was constructed by deleting nucleotides 4708–4885 between the NdeI and PflMI sites [59]. All constructs were analyzed by direct DNA sequencing prior to virus production. Virus stocks were generated by transfecting proviral DNA into 293T cells using Lipofectamine 2000 (Invitrogen) and tissue culture infectious units (TCIU) were determined using TZM-bl cells essentially as we have previously reported [24], [27], [60]. TZM-bl Hela cells and human embryonic kidney 293T cells were cultured at 37°C, 10% CO_2_ in Dulbecco's Modified Eagle Medium (Sigma) supplemented with 10% fetal bovine serum, 50 IU penicillin, 50 µg/ml streptomycin and 2 mM L-glutamine (Cellgro). CEM-SS cells were cultured at 37°C, 5%CO_2_ in RPMI 1640 (Sigma) supplemented with 10% fetal bovine serum, 50 IU penicillin, 50 µg/ml streptomycin, 2 mM L-glutamine, and 1 mM sodium pyruvate (Cellgro).

To generate a permissive cell line that can be infected with CCR5-tropic HIV, CEM-SS cells were transduced with the retroviral vector pBabe-CCR5 obtained from the NIH AIDS Research and Reference Reagent Program [61], [62]. pBabe-CCR5 and the packaging vector pEQPAM were co-transfected into 293T cells using Lipofectamine 2000 (Invitrogen). The culture supernatants were collected after 48 hours and filtered through a 0.45 µm filter. Twenty-four well plates were coated with 40 µg of Retronectin (Takara) and then washed with PBS+2% BSA and incubated twice with 0.5 mL of the vector supernatants for one hour each. CEM-SS cells (3×10^5^) were then incubated in the vector coated wells overnight at 37°C, 5% CO_2_. The following day, the vector supernatant (0.5 mL) was added to the cells overnight. Transduced cells were selected in complete RPMI containing 0.5 µg/ml puromycin. Fluorescence activated cell sorting was used to isolate the CD4^Hi^CCR5^Hi^ population with a BD FACSAria (Becton-Dickinson), collecting the top 25%.

### Viral cultures

CEM-SS cells were used to propagate both wild-type and *vif*-deficient HIV_LAI_ while CCR5 expressing CEM-SS cells were used for spreading infections with both wild-type and *vif*-deficient HIV_JR-CSF_. Cells (1×10^6^) were infected with virus stocks normalized to p24^Gag^ or tissue culture infectious units in complete RPMI containing 4 µg/ml polybrene at 37°C, 5% CO_2_ for 4 hours. The cells were washed extensively with PBS and cultured at 37°C, 5% CO_2_ in complete RPMI. Cell cultures were passaged every three days and a sample of the culture supernatant was collected for quantification of viral capsid protein by p24^Gag^ ELISA.

### APOBEC3G and APOBEC3F determination in thymocytes and CD4^+^ cells

Human CD4^+^ cells from humanized mouse spleen, liver, or lung were isolated using magnetic bead sorting (Stem Cell Technologies). A3G and A3F mRNA expression in cells was analyzed by quantitative RT-PCR (qRT-PCR) essentially as previously described [46], [47]. Briefly, cellular RNA was extracted using the RNeasy kit (Qiagen) per the manufacturer's protocol including the optional treatment with RNase-free DNase (Qiagen) during extraction. Total RNA (10 ng) was used as the template in a one-step RT-PCR reaction with the TaqMan RNA-to-Ct 1 step kit (Applied Biosystems). Primers for human A3G and A3F mRNA [47] and for human TATA Box binding protein mRNA (Applied Biosystems) were used for amplification and human A3G and A3F mRNA levels were normalized as previously described [46]. A3G protein determination was performed by disrupting cells in lysis buffer (50 mM Tris, pH = 8.0, 100 mM NaCl, 25 mM NaF, 25 mM benzamidine, 20 mM β-glycerophosphate, 2 mM Na_3_VO_2_, 3 mM EDTA, 10% glycerol, and 0.5% IGEPAL-630). Lysates were centrifuged at 13,000×g for 10 minutes and the supernatant fraction was prepared for SDS-PAGE gel electrophoresis. Separated proteins were transferred to nitrocellulose and immunoblotted for human A3G (NIH AIDS reagent program #9968) [63] and human GAPDH (Cell Signaling Technology #2118). Protein bands were quantitated by determining density using ImageJ software (Rasband, W.S., ImageJ, U. S. National Institutes of Health, Bethesda, Maryland, USA, http://rsb.info.nih.gov/ij/, 1997–2009).

### *in vivo* analysis of virus replication

Mice were maintained with the Division of Laboratory Animal Medicine at the University of North Carolina at Chapel Hill under specific-pathogen free conditions. Humanized mice (BLT and NSG-hu) were generated and analyzed for reconstitution with human hematopoietic cells including human T cells by flow cytometry essentially as previously described [20], [21], [24], [25], [27], [28]. Humanized mice were inoculated with 3×10^4^ or 9×10^4^ TCIU of wild-type HIV-1_LAI_, 9×10^4^ TCIU of wild-type HIV-1_JR-CSF_, 3.6×10^5^ TCIU of HIV_JR-CSF_Δ*vif* or HIV_LAI_Δ*vif*, or 9×10^4^ or 3.6×10^5^ HIV_JR-CSF_ *vif*FS intravenously by tail vein injection or into specific organs as indicated in the text. HIV-1 infection of humanized mice was monitored in peripheral blood by viral load analysis as previously described [27].

### Molecular analysis of HIV-1 infection

Tissues were harvested for evaluation of HIV-1 infection essentially as previously described [21]. Genomic DNA from mononuclear cells (5×10^5^–5×10^6^) from animal tissues was prepared using QIAamp DNA blood mini columns (Qiagen) according to the manufacture's protocol. Viral RNA was isolated from plasma using QIAamp viral RNA columns (Qiagen) according to the manufacture's protocol including an optional treatment with RNase-free DNase (Qiagen) during extraction and cDNA was generated using Superscript III Reverse Transcriptase (Invitrogen). Viral DNA or cDNA was amplified by nested PCR using the Expand High Fidelity PCR System (Roche).

All PCR primers amplify both HIV-1_JR-CSF_ and HIV-1_LAI_ and were designed to anneal in regions with the fewest possible putative APOBEC3 deamination sites to avoid potential primer mismatch due to APOBEC3 induced mutagenesis. HIV regions amplified include a 1.5 kb region in *pol* (RT: HIV-1_JR-CSF_ 2493–4023; HIV-1_LAI_ 2063–3595), a 1.4 kb region including *vif* and *vpr* (vif: HIV-1_JR-CSF_ 4941–6399; HIV-1_LAI_ 4511–5969), and a 900 base region in the 3′ viral genome (nef: HIV-1_JR-CSF_ 8722–9634; HIV-1_LAI_ 8328–9211). Amplification of both RT and *nef* was used to assess APOBEC3 hypermutation while *vif* was amplified to asses APOBEC3 hypermutation and to confirm the integrity (or restoration) of the ORF. Primer sequences were as follows: RT outer forward primer, GCTCTATTAGATACAGGAGC; reverse primer, CCTAATGCATATTGTGAGTCTG; RT inner forward primer, GTAGGACCTACACCTGTCAAC; reverse primer, CCTGCAAAGCTAGGTGAATTGC. Vif outer forward primer, CAGGGACAACAGAGATCC; reverse primer, GTGGGTACACAGGCATGTGTGG; vif inner forward primer, CTTTGGAA AGGACCAGCAAAGC; reverse primer, GATGCACAAAATAGAGTGGTGG. Nef outer forward primer, GAATAGTGCTGTTAGCTTGC; reverse primer, CTCAAGGCAAGCTTTATTGAGG; nef inner forward primer, TAGAGCTATTCGCCACATACC; nef inner reverse, CTTTATTGAGGCTTAAGCAGTGG. Amplified viral DNA was sequenced and compared to the corresponding proviral DNA sequence used to generate the viruses using the *Highlighter* sequence visualization tool (www.hiv.lanl.gov).

### Statistics

One-way ANOVA with Bonferroni's multiple comparison test (alpha level, 0.01), Paired two-tailed *t* tests, and Unpaired two-tailed *t* tests were all performed using Prism version 4 (Graph Pad, La Jolla, CA). All data were plotted as mean +/− SEM.

### Accession numbers

The GenBank (http://www.ncbi.nlm.nih.gov/nuccore) accession numbers for HIV-1_JR-CSF_ and HIV-1_LAI_ are M38429 and K02013. The GenPept (http://www.ncbi.nlm.nih.gov/protein) accession numbers for APOBEC3G and APOBEC3F are NP_068594 and Q8IUX4.

## Supporting Information

Figure S1 **APOBEC3F expression is reduced in the thymus.** Human A3F mRNA levels from human PBMC (n = 7), human thymus (n = 6), NSG-BLT humanized mouse thymus (n = 18), and CD4^+^ cells from NSG-BLT humanized mouse lung (n = 6), spleen (n = 7), liver (n = 7) were determined using qRT-PCR and normalized to human TATA Box binding protein. Hatched bars represent human cells; open bars represent humanized mouse cells. NS =  not significant, *** p<0.01 by one way ANOVA with Bonferroni's posttest. Data represent mean +/− SEM.
(EPS)

Figure S2 **The transient replication of CCR5-tropic HIV-1 lacking** ***vif*** **.** Plasma viral load was monitored in NSG-BLT humanized mice injected directly into the thymus with 3.6×10^5^ TCIU of HIV_JR-CSF_Δ*vif*. Viral RNA was transiently present in the blood of 2/6 mice.
(EPS)

Figure S3 **Disruption of** ***vif*** **in HIV-1_LAI_ does not affect viral replication in the absence of APOBEC3.** Spreading infection cultures with HIV_LAI_ and HIV_LAI_Δ*vif* in CEM-SS cells. Culture supernatant was assayed for p24^Gag^ by ELISA at three day intervals to determine the replication kinetics of the mutant viruses.
(EPS)
